# Inter‐rater and intra‐rater reliability of multi‐slice CT and three‐dimensional reconstructed imaging analysis of mesenteric vascular anatomy for planning and performing complete mesocolic excision

**DOI:** 10.1111/codi.70025

**Published:** 2025-03-13

**Authors:** Jordan Fletcher, Phillip Lung, Ellen Van Eetvelde, Claus Anders Bertelsen, Adam Stearns, Kristian Storli, Danilo Miskovic

**Affiliations:** ^1^ St Mark's Hospital and Academic Institute Harrow Middlesex UK; ^2^ Department of Surgery and Cancer Imperial College London London UK; ^3^ Department of Surgery University Hospital UZ Brussel Jette Belgium; ^4^ Department of Surgery Copenhagen University Hospital – North Zealand Hillerød Denmark; ^5^ Department of Clinical Medicine, Faculty of Health and Medical Sciences University of Copenhagen Copenhagen Denmark; ^6^ Department of Surgery Norfolk and Norwich University Hospitals NHS Foundation Trust Norwich UK; ^7^ Department of Surgery, Haraldsplass Deaconess Hospital University of Bergen Bergen Norway

**Keywords:** 3D, cancer, CME, colon, imaging, planning

## Abstract

**Aim:**

Complete mesocolic excision (CME) for colon cancer has been associated with improved oncological outcomes but requires a detailed understanding of complex mesenteric vasculature. Three‐dimensional (3D) reconstructed models derived from patient imaging could enhance preoperative anatomical comprehension, enabling safer, precision CME.

**Methods:**

In this two‐phase, blinded, crossover study, four expert CME surgeons evaluated mesenteric vascular anatomy on CT scans and 3D models. In phase 1, surgeons assessed 66 cases, while 20 were re‐evaluated in phase 2. The primary outcome measure was inter‐rater reliability by Fleiss's kappa. Secondary outcomes were intra‐rater reliability by Cohen's kappa and anatomical accuracy rates measured as a percentage of correct responses on a standardised questionnaire.

**Results:**

In phase 1, inter‐rater agreement was higher for 3D models (average kappa 0.6, moderate agreement) than for CT scans (average kappa 0.1, poor agreement). Ileocolic vein drainage and ileocolic artery trajectory showed the highest kappa values with 3D imaging (0.85 and 0.93, respectively). Accuracy was also superior with 3D across all surgeons (mean 89.7% correct) versus CT (mean 79.1% correct, *P* < 0.001). In phase 2, intra‐rater reliability remained higher for 3D (average Cohen's kappa 0.61) than CT scans (Cohen's kappa 0.27).

**Conclusion:**

3D mesenteric models significantly improve inter‐ and intra‐rater reliability among CME experts over traditional CT scans while markedly enhancing anatomical comprehension accuracy about critical right‐sided colonic vasculature. 3D planning could facilitate CME by enabling superior preoperative visualisation of these vessels.

**What does this paper add to the literature?**

- First validation of 3D modelling for mesenteric vascular anatomy interpretation among expert CME surgeons.
- 3D reconstructions demonstrated superior inter‐rater (*κ* = 0.6 vs. *κ* = 0.1) and intra‐rater reliability over conventional CT imaging.
- Expert anatomical interpretation accuracy increased significantly with 3D models (89.7% vs. 79.1%; *p* < 0.001).
- Critical variant detection improved substantially (95% vs. 60% for atypical ileocolic drainage), potentially enhancing surgical safety.

## INTRODUCTION

Werner Hohenberger and colleagues first introduced the concept of complete mesocolic excision (CME) for colonic cancer [1]. Central to the CME technique are three foundational principles: meticulous dissection along embryological planes, sufficient longitudinal bowel resection, and central vascular ligation coupled with the excision of central aspects of the mesocolon [2]. CME represents an advanced surgical procedure demanding a high level of technical skill. A key challenge inherent to CME is navigating the complex mesenteric vascular anatomy, particularly for lesions in the right and transverse colon [2].

The central dissection performed in CME contains a risk of vascular injury, which has raised concerns about the safety of adopting this technique widely beyond high‐volume expert centres [3, 4]. Results regarding the risk of vascular injury reported in two recent randomised trials are contradictory [5, 6]. However, the central dissection does not appear to be associated with an overall higher morbidity and mortality [7, 8].

A detailed understanding of colonic and mesenteric anatomical variations is essential for preoperative planning and execution of these extensive surgical procedures [9]. Traditionally, this understanding is acquired by examining CT datasets. However, translating the three‐dimensional (3D) anatomical relationships inferred from two‐dimensional (2D) images for intraoperative application presents a significant challenge, even for experienced surgeons [10]. Moreover, clinically relevant anatomical variations are often underreported.

The advent of 3D reconstructed models from patient imaging data has the potential to enhance anatomical comprehension [11], serving as a valuable tool for surgical planning and execution [12]. These 3D reconstructions, theoretically, offer advantages over conventional 2D imaging, yet their utility and reliability in mesenteric vessel visualisation have yet to be empirically validated in an expert audience [13, 14].

This study aims to assess the intra‐ and inter‐rater reliability of multi‐slice CT imaging versus 3D reconstructed models among colorectal surgeons with expertise in performing CME.

## METHODS

### Study aims

The study's objectives are twofold: first, to evaluate the inter‐ and intra‐rater reliability of 3D models compared to standard CT scans in the assessment of mesenteric blood vessel configurations pertinent to CME, and second, to ascertain the accuracy of each imaging modality in this context.

### Outcome measure

The primary outcome measure was an objective vascular anatomy knowledge questionnaire (File S1). The questionnaire was developed by the research team to assess the understanding of the mesenteric vascular anatomy in a clinical context relevant to performing right‐sided CME.

### Participant selection and data collection

Four expert CME surgeons were recruited for the study, three of whom serve as instructors for the European Society of Coloproctology minimally invasive training course [9], with the fourth having extensive experience performing CME. We collected surgeon CME experience and typical preoperative planning processes.

**FIGURE 1:**
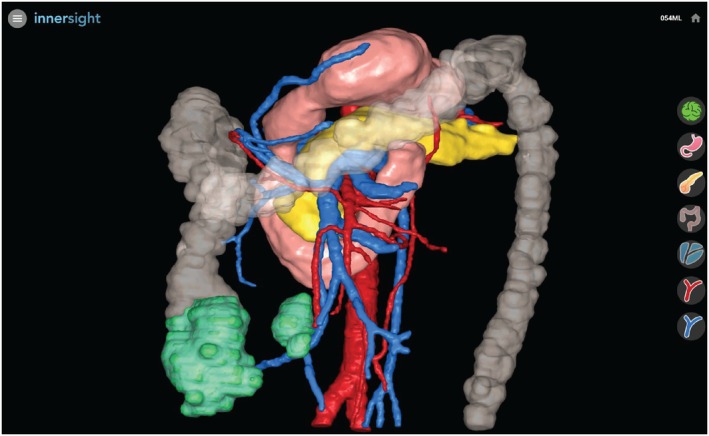
3D surface rendered reconstructed model with associated web‐based user interface [17].

Sixty‐six patient cases were randomly selected for analysis from a prospectively maintained CME database containing 100 total reconstructed scans by using a random number generator to create a sequence from 1 to 100, the first 66 unique numbers pulled corresponding to included cases. 3D reconstructions of these cases were created by manually segmenting (JF) each CT scan using ITK SNAP software (version 3.8, open‐source) [15, 16], and were validated by a consultant gastrointestinal radiologist (PL). The time spent segmenting each scan was recorded to the nearest 5 min. 3D polygon mesh models were generated from segmentation files using a marching cubes algorithm [17], with subsequent mesh optimisation through decimation (reducing polygon count while preserving shape) and Laplacian smoothing (minimising stair‐step artefacts caused by discrepancies between image slices, resulting in a more natural appearance) to balance visual fidelity and render efficiency [18, 19]. The resulting models, coloured according to the standard anatomical colour scheme, were integrated into a web‐based interface powered by Three.js, enabling interactive exploration with features such as rotation, zooming and selective transparency of individual mesh components (see Figure 1) [20, 21].

Reconstructions were verified by JF and PL from intraoperative video footage from a prospectively maintained CME database.

Before the study, participating surgeons were provided with a comprehensive manual detailing the definitions of mesenteric vessels (File S2) and a tutorial on utilising the 3D models. To ensure blinding, scan data and 3D models for each case were encoded with a random identifier, preventing participants from associating models with corresponding CT scans.

### Testing procedure and analysis

The study comprised two rounds of testing. In the first round, participants completed a multiple‐choice anatomical questionnaire for each of the 66 cases, first using CT scan data and then 3D models, and vice versa, totalling 132 assessments. A 3‐month washout period followed before the second round, which involved a random selection of 20 cases from the initial 66. Participants repeated the questionnaire for these 20 cases using 3D models and CT imaging, each re‐encoded to maintain blinding from the first round.

Responses were electronically captured using Microsoft Forms (Microsoft Corp. 2023).

### Data analysis

Data analysis was conducted using Microsoft Excel (Microsoft Corp. 2023) and custom Python 3.4 scripts within a Jupyter Notebook environment [22], offering a reproducible and transparent computational framework. Data manipulation and analysis were facilitated by specific Python libraries: Numpy [23] and Pandas [24] for data handling, Matplotlib [25] and Seaborn [26] for visualisation, and SciPy [27] and Stats for statistical analysis. To assess the inter‐rater reliability in round 1, we calculated the Fleiss kappa value for each single‐response answer, chosen for its appropriateness in evaluating agreement across multiple raters. For multi‐response items, responses were initially encoded using a one‐hot encoding scheme, enhancing data compatibility for subsequent analysis. We then computed the mean kappa value for each question to provide a measure of consensus amongst raters.

Kappa results were interpreted as follows: values ≤0 as indicating no agreement and 0.01–0.20 as none to slight, 0.21–0.40 as fair, 0.41–0.60 as moderate, 0.61–0.80 as substantial and 0.81–1.00 as almost perfect agreement [28].

The average agreement was quantified as the percentage of similar responses given by each rater for every case per question, offering insights into the consistency of raters' judgements. Similarly, the intra‐rater reliability for each imaging modality, including CT and 3D models, was determined using Cohen's kappa. This was complemented by calculating the average agreement, again expressed as the percentage of matching responses.

We calculated the average number of correct anatomical identifications per case for each participant. The benchmarks for correct anatomy in each case were established through previous intraoperative observations in combination with review of the imaging by a gastrointestinal radiologist with expertise in CME anatomical evaluation (PL). Owing to the non‐normal distribution of our data, confirmed by the Shapiro–Wilk test, we applied non‐parametric statistical techniques for analysis. The Mann–Whitney *U* test was specifically utilised to compare the accuracy of anatomical identifications between 3D reconstructed models and multi‐slice CT scans.

### Sample size determination

The study analysed data from 66 patient cases. The sample size was calculated based on an estimated kappa value of 0.7, which falls within the ‘good’ agreement range of 0.6–0.8, providing a confidence interval of ±0.12.

## RESULTS

### Participating surgeon demographics

The CME operative and imaging interpretation experience and preoperative planning practices of each participating surgeon are outlined in Table 1.

**TABLE 1:** CME experience and preoperative planning practices of participating surgeons.

Surgeon	CB	EVE	AS	KS
Operative experience				
Number of years performing CME	16	5	8	17
Number of CMEs performed	700	120	400	1100
Open	250	0	20	100
Laparoscopic	450	0	380	1000
Robotic	0	120	0	0
Preoperative planning processes and imaging interpretation experience				
Reviews CT imaging preoperatively to determine vascular anatomy	Yes, alone	Yes, alone	Yes, alone	Yes, alone
Does radiology report contain any information regarding anatomical variants?	No	No	No	No
Formal training in CT interpretation for CME anatomy?	Taught on course	None	Taught on course	Taught on course
Do you use 3D reconstructed models to plan and perform CME?	No	No	No	No

Abbreviation: CME, complete mesocolic excision.

### Segmentation time

Table 2 presents the segmentation times for each type of scan, while Figure S1 shows how these times were distributed across the dataset.

**TABLE 2:** Segmentation times summary as per scan protocol and slice thickness.

Scan category	*n*	Time (min)				Total segmentation time (min)
		Median	IQR	Min	Max	
CT PV contrast (slice thickness ≤1 mm)	4	140	3.75	135	150	565
CT PV contrast (slice thickness >1 to ≤2.5 mm)	47	75	25	50	100	3600
CT PV contrast (slice thickness >2.5 mm)	4	57.5	7.5	50	65	230
CT colon	11	145	20	125	175	1615

Abbreviations: IQR, interquartile range PV; portovenous.

### Inter‐rater reliability

The Fleiss's kappa statistical analysis (Table 3) conducted for round 1 inter‐rater reliability across 66 cases demonstrated a substantially better agreement for most anatomical landmarks when the 3D models were used. The overall Fleiss's kappa was 0.6 for 3D and 0.1 for CT.

**TABLE 3:** Comparative inter‐rater reliability measured by Fleiss's kappa for CT and 3D imaging modalities during round 1.

Question	CT	3D
ICV drainage	0.13	0.85
ICA trajectory	0.56	0.93
RCA present?	0.02	0.79
RCA trajectory	0.02	0.75
RCV present?	0.04	0.25
RCV drainage	0.03	0.32
No. of MCA trunks	0.01	0.47
Origin of MCA	0.15	0.75
No. of MCV trunks	0.10	0.18
MCV drainage	0.25	0.43
Constituent vessels of Henle	0.14	0.50
Henle class	0.14	0.62
No. of ASPDV	0.05	0.59
ASPDV drainage	0.08	0.73
First jejunal vein trajectory	0.30	0.57

*Note*: This table enumerates the Fleiss kappa statistics for each anatomical question, assessing the agreement between raters when utilising CT and 3D imaging techniques. Colour key for Fleiss's kappa values: red, *κ* = 0 to <0.2 (poor agreement); orange, *κ* = 0.21–0.40 (fair agreement); yellow, *κ* = 0.41–0.60 (moderate agreement); light green, *κ* = 0.61–0.80 (good agreement); green, *κ* = 0.81–1.00 (very good agreement).

Abbreviations: ASPDV, anterior superior pancreatico‐duodenal vein; ICA, ileocolic artery; ICV, ileocolic vein; MCA, middle colic artery; MCV, middle colic vein; RCA, right colic artery; RCV, right colic vein.

### Intra‐rater reliability

The intra‐rater reliability for CT and 3D imaging modalities, as measured by Cohen kappa values, demonstrated a range of agreement across anatomical structures assessed. The average Cohen kappa values were higher for 3D when compared to CT, as shown in Table 4.

**TABLE 4:** Intra‐rater reliability assessment using Cohen's kappa for CT versus 3D imaging across anatomical questions.

Intra‐rater Cohen kappa values for CT and 3D image modalities										
Question	Rater 1		Rater 2		Rater 3		Rater 4		Average Cohen kappa	
	CT	3D	CT	3D	CT	3D	CT	3D	CT	3D
ICV drainage	0.0	0.6	1.0	1.0	0.5	0.6	0.0	0.8	**0.37**	**0.76**
ICA trajectory	0.9	0.8	0.7	1.0	0.7	0.9	0.5	1.0	**0.68**	**0.92**
RCA present?	0.2	0.6	0.4	0.5	0.1	0.7	0.0	0.7	**0.18**	**0.63**
RCA trajectory	0.1	0.6	0.4	0.5	0.0	0.7	0.0	0.6	**0.13**	**0.62**
RCV present?	0.6	0.2	0.3	0.7	0.0	0.2	0.0	0.4	**0.22**	**0.37**
RCV drainage	0.7	0.3	0.4	0.7	0.0	0.2	0.2	0.4	**0.32**	**0.42**
No. of MCA trunks	0.0	0.6	0.0	0.6	0.0	0.5	0.1	0.7	**0.03**	**0.59**
No. of MCV trunks	0.0	0.2	0.4	0.4	0.3	0.2	0.3	1.0	**0.25**	**0.46**
Henle class	0.1	0.4	0.2	0.9	0.1	0.8	0.2	0.7	**0.17**	**0.69**
ASPDV	0.0	0.7	0.4	0.8	0.3	0.7	0.4	0.7	**0.30**	**0.70**
First jejunal vein trajectory	0.3	0.7	0.4	0.7	0.4	0.8	0.5	0.1	**0.39**	**0.56**

*Note*: This table quantifies the consistency of each rater's responses between rounds 1 and 2 by presenting Cohen's kappa values for both CT and 3D imaging modalities. Colour key for Fleiss's kappa values: red, *κ* = 0 to <0.2 (poor agreement); orange, *κ* = 0.21–0.40 (fair agreement); yellow, *κ* = 0.41–0.60 (moderate agreement); light green, *κ* = 0.61–0.80 (good agreement); green, *κ* = 0.81–1.00 (very good agreement).

Abbreviations: ASPDV, anterior superior pancreatico‐duodenal vein; ICA, ileocolic artery; ICV, ileocolic vein; MCA, middle colic artery; MCV, middle colic vein; RCA, right colic artery; RCV, right colic vein.

### Accuracy

In a comparative analysis of diagnostic accuracy, 3D imaging was consistently associated with higher correct diagnosis rates across all evaluators compared to CT imaging.

**FIGURE 2:**
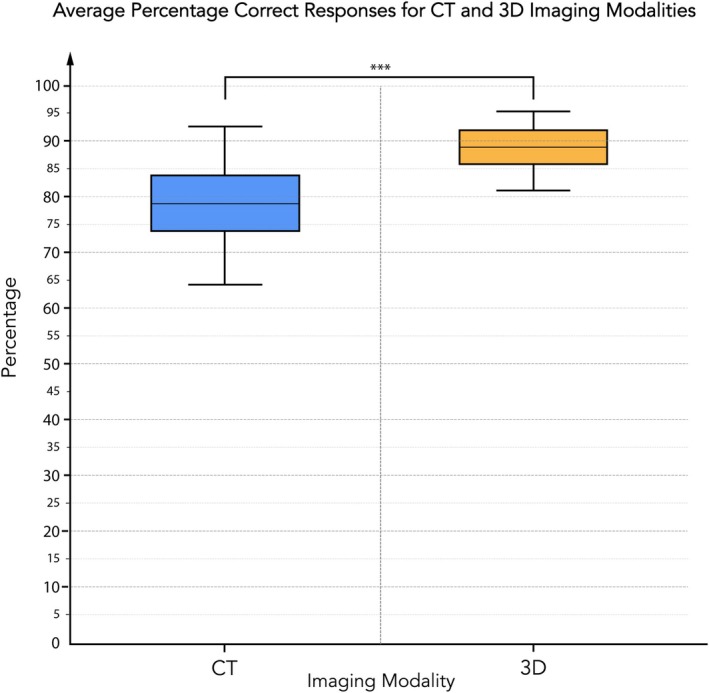
Box plot analysis of average correct response rates by imaging modality. This box plot contrasts the distribution of the average percentage of correct responses for each rater using CT (shown in blue) and 3D imaging modalities (shown in orange). The central line in each box represents the median percentage, the box itself shows the interquartile range and the whiskers extend to the range of data points.

In round 1, in which 66 cases were evaluated, the mean percentage of correct responses for CT ranged from 76% to 85%, while for 3D imaging the mean percentages were higher, spanning from 87% to 93% (see Table 5 and Figure 2). The second round, with 20 cases, showed a similar trend, with mean correct responses for CT ranging from 78% to 83% and for 3D from 87% to 90% (Table 5). Box and whisker plots depicting the distribution of correct response percentages for each rater are provided in Figure S2.

**TABLE 5:** Mean percentage of correct responses for CT and 3D imaging modalities by rater.

	CT correct responses (%)				3D correct responses (%)				*P* value
	Mean	Min	Max	SD	Mean	Min	Max	SD	
Round 1, *n* = 66									
Rater 1	**79**	49	97	11.7	**90**	68	100	7.5	*P* < 0.001
Rater 2	**85**	68	100	8.7	**92**	68	100	7.9	*P* < 0.001
Rater 3	**76**	49	97	11.8	**93**	76	100	6.0	*P* < 0.001
Rater 4	**77**	51	97	9.7	**87**	60	100	10.0	*P* < 0.001
Round 2, *n* = 20									
Rater 1	**81**	70	100	8.2	**87**	70.3	100	7.2	*P* < 0.001
Rater 2	**83**	62	97	9.1	**90**	78.4	100	5.8	*P* < 0.001
Rater 3	**80**	62	97	12.0	**88**	51.4	100	11.7	*P* < 0.001
Rater 4	**78**	65	100	9.2	**87**	67.6	100	9.5	*P* < 0.001

Minimum correct response rates for the first round for CT varied between 49% and 68%, with 3D imaging at 60%–76%. In the second round, the minimum percentages increased slightly for CT, ranging from 62% to 70%, and for 3D from 51% to 78%. Maximum correct response rates in both rounds reached as high as 100% for both modalities.

The standard deviation of correct responses for 3D imaging was consistently lower than that for CT in both rounds, indicating more consistent ratings among the raters when using 3D imaging. Statistically significant differences between the modalities were confirmed with *P* values less than 0.001 for all raters in both rounds.

### Performance of 3D models relative to CT imaging by case and rater

**FIGURE 3:**
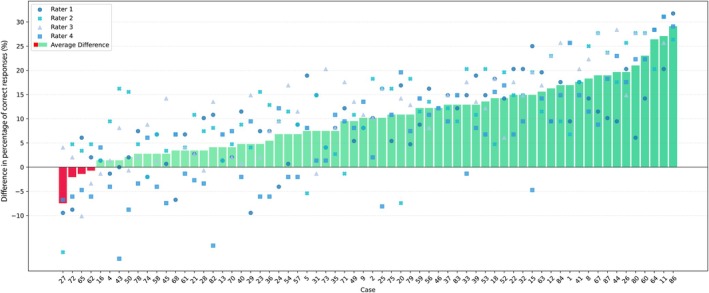
Differential rater performance in correct responses using CT imaging versus 3D models for round 1. The bar chart illustrates the average absolute percentage difference in correct responses for each case, with the green bars representing the aggregated data. The overlaid scatter plot depicts individual rater performance, with each rater denoted by a unique colour and symbol.

In the first round of case assessments, use of the 3D virtual models conferred improved diagnostic accuracy compared to CT scans alone in 62 out of the total 66 cases (94%). The median accuracy improvement was 10.1 percentage points across all raters (interquartile range 10.8%). However, gains varied widely from −7.4 points (worse than CT) up to 29.1 average percentage points in individual cases (Figure 3).

**FIGURE 4:**
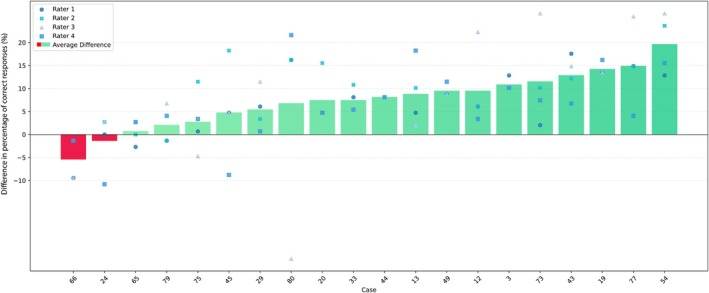
Differential rater performance in correct responses using CT imaging versus 3D models for round 2. The bar chart illustrates the average percentage difference in correct responses for each case, with the green bars representing the aggregated data. The overlaid scatter plot depicts individual rater performance, with each rater denoted by a unique colour and symbol. Figure S4 presents line plots for each rater, depicting the percentage of correct responses per case for both 3D and CT modalities in round 2.

In the 20 cases evaluated in the second round, 3D reconstructed models enhanced accuracy over CT in 18 out of 20 cases (90%). The median improvement decreased to 7.8 percentage points (interquartile range 8.5 points) with a reduced span from −5.4 points to 19.6 points (Figure 4).

When analysed by surgeon, median accuracy benefits ranged from 6.1 to 17.9 percentage points (round 1) and from 4.8 to 10.5 points (round 2). But despite high averages, interquartile ranges demonstrating substantial variability persisted both between surgeons and across cases in the improvements afforded by 3D model use.

Raters 1–4 achieved higher accuracy with CT scans in 12%, 17%, 8% and 14%, respectively.

Figures S3 and S4 present line plots for each rater, depicting the percentage of correct responses per case for both 3D and CT modalities in rounds 1 and 2 respectively.

### Accuracy by mesenteric vessel

Evaluating the correct response percentages by question for each rater using CT and 3D imaging modalities revealed that 3D imaging consistently yielded higher correct response rates compared to CT for most anatomical features assessed (Table 6). The Mann–Whitney *U* test confirmed a significant difference in rater accuracy between CT and 3D modalities, with a *U* statistic of 62.0 and a *P* value of 0.038.

**TABLE 6:** Number (*n*) and percentage correct responses (%) for each anatomical questionnaire item for CT and 3D.

Correct response percentage for each rater using CT and 3D imaging modalities																		
Question	Rater 1				Rater 2				Rater 3				Rater 4				% average correct responses	
	CT		3D		CT		3D		CT		3D		CT		3D			
	*n*	%	*n*	%	*n*	%	*n*	%	*n*	%	*n*	%	*n*	%	*n*	%	CT	3D
ICV drainage	60	91	64	97	66	100	65	98	53	80	65	98	58	88	65	98	**89.8**	**98.1**
ICA trajectory	58	88	64	97	66	100	66	100	55	83	66	100	51	77	64	97	**87.1**	**98.5**
RCA present?	55	83	64	97	54	82	65	98	38	58	64	97	50	76	59	89	**74.6**	**95.5**
RCA trajectory	55	83	61	92	52	79	63	95	36	55	62	94	47	71	57	86	**72.0**	**92.0**
RCV present?	29	44	20	30	22	33	13	20	15	23	17	26	18	27	23	35	**31.8**	**27.7**
RCV drainage	30	45	46	70	44	67	51	77	36	55	48	73	41	62	43	65	**57.2**	**71.2**
No. of MCA trunks	59	89	62	94	52	79	59	89	47	71	61	92	45	68	61	92	**76.9**	**92.0**
Origin of MCA	65	99	66	100	66	99	66	100	65	98	66	100	65	99	65	98	**98.8**	**99.6**
No. of MCV trunks	35	53	45	68	39	59	45	68	29	44	46	70	30	45	37	56	**50.4**	**65.5**
MCV drainage	53	80	58	88	57	87	60	90	54	82	61	92	54	81	57	86	**82.5**	**89.0**
Constituent vessels of Henle	51	78	57	87	54	82	60	91	51	77	61	92	51	77	56	85	**78.6**	**89.0**
Henle class	30	45	54	82	38	58	57	86	32	48	57	86	29	44	46	70	**48.9**	**81.1**
No. of ASPDV	23	35	38	58	38	58	44	67	34	52	41	62	28	42	36	55	**46.6**	**60.2**
ASPDV drainage	50	76	61	93	51	77	62	94	51	77	64	96	43	66	60	91	**73.9**	**93.8**
First jejunal vein trajectory	50	76	54	82	56	85	60	91	45	68	61	92	46	70	46	70	**74.6**	**83.7**

Abbreviations: ASPDV, anterior superior pancreatico‐duodenal vein; ICA, ileocolic artery; ICV, ileocolic vein; MCA, middle colic artery; MCV, middle colic vein; RCA, right colic artery; RCV, right colic vein.

Correct response rates for individual items are shown in paired bar charts (Figure S5), with detailed stratification by anatomical variation in Table S1. Figure S6 illustrates the distribution of correct responses across anatomical variants, while Figure S7 presents box and whisker plots comparing imaging modalities for Henle's trunk vessel configurations.

## DISCUSSION

### Summary and key findings

The current study examines the inter‐rater and intra‐rater reliability of 3D reconstructed models versus multi‐slice CT scans in the context of mesenteric vascular mapping for CME. 3D models offer a statistically significant improvement in the consistency of anatomical interpretations among different expert raters and for the same rater over time. Furthermore, we observed improved accuracy of assessments when raters employed 3D models, especially in the identification and interpretation of complex anatomical landmarks. These results suggest potential for integrating 3D reconstruction into standard preoperative planning for CME.

### Interpretation of results

The enhanced anatomical understanding provided by 3D visualisation is evident for vital CME structures such as the ileocolic artery and vein, components of Henle's trunk, and the middle colic vessels. Although 3D models generally enhanced anatomical understanding for each evaluator, this improvement was not consistent across all cases assessed. This suggests that the utility of 3D models is superior yet not absolute and may vary depending on the specific clinical scenario. Therefore, surgeons' expertise in interpreting both 3D reconstructions and traditional imaging continues to be indispensable, with 3D models serving as a complementary tool in the surgical planning process.

3D models demonstrate superior inter‐rater reliability as assessed using Fleiss's kappa values. For clinically relevant features pertinent to CME, 3D imaging yielded substantial to almost perfect agreement among raters, while CT imaging often fell into the low agreement range. This suggests that 3D models not only improve agreement among different raters but also enhance the consistency of individual rater assessments over time.

The literature on 3D imaging in operative planning, especially concerning mesenteric anatomy, is evolving but largely limited to early technical/feasibility work [13, 29]. This study contributes robust evidence supporting the superior anatomical understanding afforded by 3D models. Furthermore, it complements existing research, such as our previous work on trainee surgeons [12] and research validating 3D reconstruction in the spatial configuration of mesenteric branches [13, 29].

### Clinical implications

The enhanced accuracy and reliability of 3D imaging in visualising the mesenteric vasculature hold significant clinical implications, especially for complex procedures like CME. While our study demonstrates statistically significant median accuracy improvements of 10.1% in phase 1 and 7.8% in phase 2 when using 3D models, it is crucial to illustrate how these enhancements translate into tangible surgical benefits.

**FIGURE 5:**
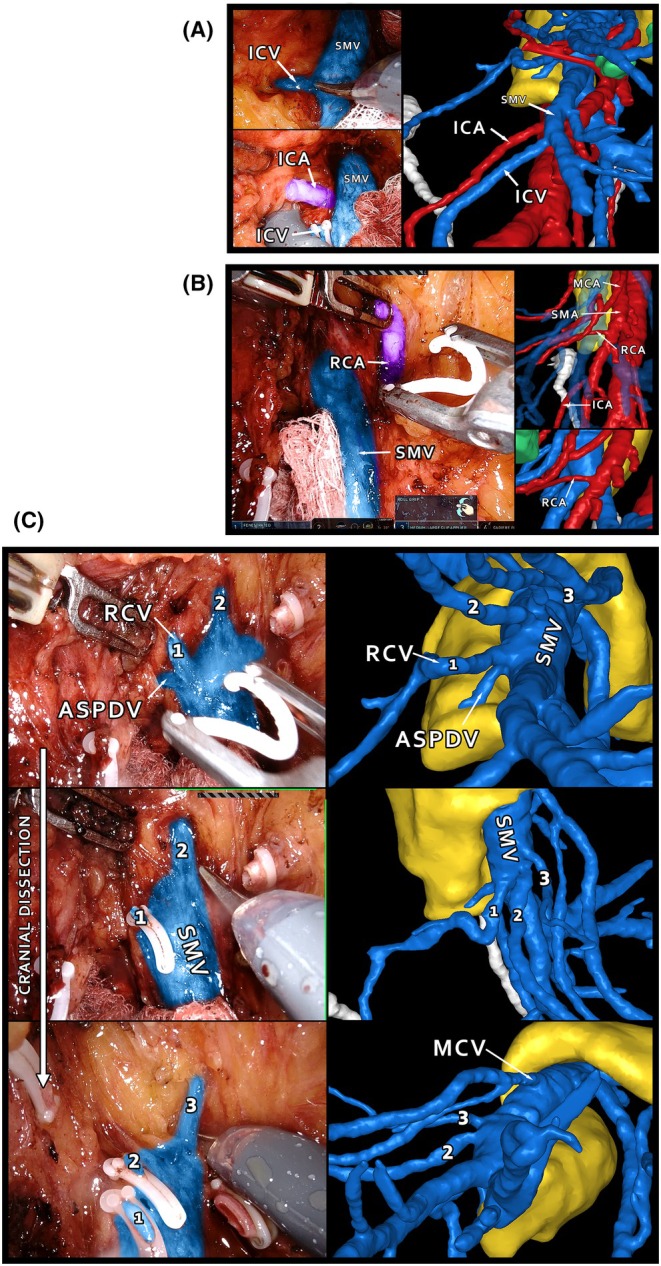
(A–C) Comparative view of intraoperative findings compared with 3D reconstruction for a CME case with three separate colonic veins that were found to drain ICV directly into SMV. ASPDV, anterior superior pancreatico‐duodenal vein; ICA, ileocolic artery; ICV, ileocolic vein; MCA, middle colic artery; RCA, right colic artery; RCV, right colic vein; SMV, superior mesenteric vein.

One of the key advantages of 3D imaging is its ability to facilitate the recognition of rare anatomical variants that can significantly impact surgical planning and execution. For instance, in our study, five cases exhibited an atypical drainage pattern where the ileocolic vein drained directly into Henle's trunk instead of the superior mesenteric vein (SMV). Participants correctly identified this variant in 95% of cases (19 out of 20) using 3D imaging, compared to only 60% with conventional CT. Notably, one rater failed to recognise this variant in any case using CT. The ileocolic vein is a crucial anatomical landmark, and its ligation is one of the early critical steps in CME [30]. If the vein drains into Henle's trunk, it may not be encountered during the usual portion of the operation, potentially leading to unexpected bleeding or ligation of the SMV or terminal ileal branches mistaken for the ileocolic vein. Figure 5 demonstrates an unusual anatomical variant identified through 3D reconstruction, showing three separate middle colic veins draining independently into the SMV. Preoperative recognition of such variants through 3D imaging can thus prevent iatrogenic injury, reduce operative time and enhance the surgical team's confidence.

Similarly, 3D imaging shows a significant advantage in identifying smaller calibre vessels like the right colic artery. Defined as an independent branch originating from the superior mesenteric artery between the middle and ileocolic arteries, the right colic artery was present in eight cases. All raters correctly identified it in 97% of cases (35 out of 36) using 3D imaging, compared to only 34% (11 out of 36) with CT. Recognising the presence of a right colic artery preoperatively is crucial because it can alter the surgical approach. Unanticipated encounter with this vessel during surgery can lead to intraoperative complications such as bleeding or may necessitate a change in strategy, affecting both the safety and efficiency of the procedure. Similarly, models can help recognise potentially dangerous variants such as a jejunal vein passing caudally to the origin of the middle colic artery. In a video abstract we demonstrate how models can help not only recognise this variant but help use it to target dissection and facilitate intraoperative navigation [31].

3D models provide a comprehensive understanding of the spatial relationships between vascular structures, which is not easily appreciated on conventional CT scans. For example, Figure 5 depicts a case with three separate colonic veins inserting into the SMV. Recognition of more than one middle colic vein was extremely poor using CT scan (3%–29% correct identification; see Figure S5). The 3D models not only help identify the presence of each vessel but also offer insights into their relative positions and trajectories and avoid injury and subsequent bleeding directly from the SMV. This spatial appreciation is vital during CME to avoid vascular injuries and ensure complete lymphadenectomy.

While the median accuracy improvements were statistically significant, they were not universal across all cases. This raises the question of how much these improvements in anatomical understanding correspond to clinical benefits. It is important to acknowledge that our assessment focused on specific facets of anatomical understanding—namely, the presence of a vessel, its origin or drainage pattern. This does not capture other critical components such as scale, relative location and the spatial relationships between structures. Therefore, we may not be fully appreciating the qualitative benefits that 3D models provide in fostering a more holistic understanding of mesenteric anatomy.

Translating enhanced anatomical visualisation into better patient outcomes is complex and influenced by multiple factors, including variations in surgical technique, individual surgeon experience, intraoperative decision‐making and patient‐specific considerations. While our study provides evidence that 3D imaging improves anatomical identification, proving that this leads to reduced vascular injuries, decreased operation times or enhanced surgical confidence requires further investigation. Such translational studies would necessitate large sample sizes and multi‐institutional collaboration to achieve the statistical power needed for definitive conclusions, which may be challenging to obtain. Comparable studies using 3D to perform liver resections and partial nephrectomy have demonstrated that the use of 3D models significantly reduces blood loss and operative time [5, 7, 32].

Despite these limitations, our findings suggest that 3D imaging enhances agreement and accuracy concerning critical structures integral to performing safe CME. Structures such as the drainage pattern of the ileocolic vein, the trajectory of the ileocolic artery, the number and origin of the middle colic arteries, and the number and drainage patterns of the middle colic veins are pivotal for surgical planning and execution. While it is difficult to quantify precisely how many CMEs transition from ‘unsafe’ to ‘safe’ based solely on our study, the notable improvements in identifying these critical structures with 3D models indicate a potential for significant surgical and technical benefits. By pre‐emptively recognising anatomical variations, surgeons can tailor their approach to each patient, potentially reducing the risk of intraoperative complications and improving overall surgical outcomes.

### Study limitations

Our study has several limitations that warrant consideration. First, despite our efforts to provide precise anatomical definitions, subjectivity in vessel classification may not have been eliminated. Incorrect responses from raters might stem from misclassification rather than a genuine lack of understanding. This issue is particularly evident with vessels such as the middle colic vein, right colic vein and superior right colic vein. Raters may identify a draining vein in regions like the ascending colon, hepatic flexure or transverse colon, but variations in interpretation and naming conventions can lead to discrepancies. For example, differentiating between a superior right colic vein and a right colic vein can be challenging, potentially contributing to the observed low accuracy in identifying the right colic vein on 3D scans.

Including only four experienced CME surgeons as raters introduces the possibility of expertise bias, potentially limiting the generalizability of our findings to less specialised surgeons. However, in previous work, we demonstrated similar improvements in anatomical understanding with 3D models among trainees with limited CME experience [12]. While the magnitude of improvement and overall accuracy may differ without CME subspecialisation, 3D models appear to enhance anatomical comprehension regardless of baseline expertise.

The study assumes uniform high quality in both the 3D reconstructed models and CT scans across all cases. Variability in image acquisition and reconstruction quality can influence the accuracy of anatomical interpretation, potentially confounding the results. Additionally, having the same team responsible for both the 3D reconstruction and the definition of the gold standard introduces potential bias. This dual role could inadvertently align the gold standard more closely with our 3D models than if an independent team had set the standard. While we believe this bias is minimal—since all 3D models were reviewed by a consultant gastrointestinal radiologist and validated using intraoperative findings—we acknowledge its potential influence on our results. Future studies should consider involving independent teams for reconstruction and validation to enhance robustness.

A significant practical limitation is the time‐intensive nature of manual segmentation required to generate the 3D models. Our findings reveal that even experienced segmenters using dedicated equipment require a median time of 49–136 min per case, depending on imaging modality and slice thickness. This substantial time investment poses challenges for integration into busy clinical workflows, where rapid turnaround is essential. However, given the variability of colonic anatomy, we advocate the use of 3D models in all CME cases. Even when the anatomy appears ‘typical’, surgeons can derive significant benefits from a better appreciation of spatial relationships during intraoperative dissection.

While we demonstrate in principle the superiority of 3D, the reliability and accuracy of these reconstructions depend wholly on the quality of segmentation and software rendering. Benefits to anatomical understanding are therefore contingent on the validity of the reconstruction. We reduced this risk by reviewing all 3D models with a consultant gastrointestinal radiologist and validated each model using intraoperative findings.

The statistical measures used to evaluate inter‐rater and intra‐rater reliability have their limitations. Fleiss kappa and Cohen kappa values are sensitive to the prevalence of the attribute being measured and the bias of raters, which can lead to paradoxical situations where observed agreement is high but kappa values are low. This phenomenon, known as the kappa paradox, can result in misinterpretation of the agreement levels among raters [33]. However, the level of average intra‐rater agreement observed corroborates our findings and overall conclusions.

### Practical considerations for 3D reconstruction

Implementing 3D reconstructions routinely would require significant adjustments in workflow and resource allocation. While radiologists possess expertise in image interpretation, the creation of detailed, interactive 3D reconstructions with colour coding and selective transparency may demand additional training and resources not commonly available in radiology departments. This raises the question of whether surgeons should become more proficient in generating these models or if the task should be delegated entirely to radiologists or specialised technicians. In most clinical settings, radiologists are responsible for imaging tasks, while surgeons focus on translating imaging data into surgical plans. A collaborative approach may be the most practical solution, leveraging the strengths of both disciplines to ensure that 3D models are produced efficiently and accurately.

Manual segmentation also introduces an inherent lack of reproducibility. The accuracy of reconstructions heavily relies on the subjective interpretation of imaging data by the individual performing the segmentation. This variability underscores the need for more standardised and objective approaches. To overcome these laborious processes, exploring alternative solutions is crucial. Semi‐automated segmentation tools that leverage artificial intelligence (AI) assistance while retaining clinician oversight could offer a balance between efficiency and accuracy [34].

Despite impressive advances in AI‐driven automated segmentation systems [35, 36], no fully automated algorithms currently exist for mesenteric vessel segmentation from standard CT scans [37]. To establish best practices and identify optimal solutions, comprehensive benchmarking studies are needed to compare the performance of different model architectures on large, well‐annotated datasets [38].

Previous attempts at semi‐automated vessel segmentation, such as the modified vesselness and active growing segmentation (MVAGS) algorithm described by Luzon et al. [39], have limitations. Their approach produced a single mesh representation of the mesenteric vasculature without the ability to apply colour coding or selective transparency to individual vessels, and it did not include contextual organ anatomy. We feel such interactive visual elements are crucial for enhancing comprehension and maximising the utility of 3D vascular models in clinical practice.

Advancements in AI offer a promising path to overcome these limitations. The learning process involves a cycle of prediction and correction. The model makes an initial guess based on the input data, compares this guess to the known correct answer (often referred to as the ‘ground truth’) and then adjusts its internal calculations to improve its accuracy. This process repeats many times, gradually refining the model's ability to make accurate predictions [40].

Deep learning architectures like convolutional neural networks and transformers have shown potential in medical image segmentation tasks [41]. However, the development and validation of AI‐driven segmentation tools face several challenges.

1. Data requirements: Training robust AI models necessitates large, high‐quality datasets of manually segmented scans, which are often difficult and time‐consuming to acquire.
2. Computational demands: Processing large volumes of CT data for AI model training requires significant computational resources, in terms of both hardware capabilities and the requisite associated financial investment.
3. Accuracy assessment: For tasks like organ or vessel segmentation, model performance is often measured using metrics such as the Dice coefficient and Jaccard index. These measure how well the model's segmentation aligns with expert‐created segmentations, scoring from 0 (no overlap) to 1 (perfect alignment) [42]. However, these metrics may not fully capture clinical relevance. A model could achieve high scores overall but still miss small, clinically crucial details like tiny blood vessels. While statistically minor, such errors could significantly impact surgical planning.

To minimise the laborious processes currently required, investing in the development of fully automated, algorithm‐driven segmentation tools is essential. Such tools could dramatically streamline the creation of 3D models, making them more accessible for routine clinical use. Until then, outsourcing segmentation to specialised services with expertise in medical image analysis could provide an interim solution, allowing clinical teams to focus on core responsibilities without the significant time burden.

## CONCLUSIONS AND AREAS FOR FURTHER STUDY

Our study highlights the potential advantages of 3D reconstructed models in enhancing anatomical understanding of mesenteric vascular anatomy during CME surgery. While the statistically significant improvements in accuracy are promising, the clinical impact of these gains requires further validation through larger studies. Nonetheless, our findings support the integration of 3D reconstructions into routine CME practice. The superior anatomical visualisation provided by 3D models can aid in preoperative planning and intraoperative navigation when used alongside standard CT scans.

To build upon the current work, several areas merit further exploration. First, feasibility studies are essential to assess the practical integration of 3D models into CME surgery. These studies should evaluate how 3D reconstructions affect surgical workflows, decision‐making processes and the learning curve associated with CME procedures. Gathering subjective feedback from surgeons will offer valuable insights into the usability and perceived benefits of 3D models, potentially guiding improvements in their application. Long term large‐scale, multicentre trials would be instrumental in establishing definitive evidence of their benefits.

Additionally, efforts should be directed toward developing and validating automated segmentation algorithms. AI‐driven solutions, particularly those leveraging deep learning, have the potential to significantly streamline the generation of 3D models, reducing the time burden on clinical teams. Benchmarking studies comparing different segmentation models on well‐annotated datasets would help establish best practices for mesenteric vessel segmentation.

Finally, the practical integration of 3D models into routine surgical workflows requires investigation. Studies should evaluate the cost‐effectiveness, resource implications and learning curve associated with their adoption. Research into the collaborative roles of radiologists, surgeons and specialised technicians in generating and utilising these models will help determine the most efficient and effective approach for widespread implementation. By addressing these gaps, future research can help ensure that the benefits of 3D modelling extend beyond theoretical advantages and translate into tangible improvements in patient care.

## AUTHOR CONTRIBUTIONS

**Jordan Fletcher:** Conceptualization; investigation; methodology; formal analysis; data curation; visualization; writing – original draft; software; project administration; writing – review and editing. **Phillip Lung:** Conceptualization; methodology; formal analysis; writing – review and editing; validation; supervision. **Ellen Van Eetvelde:** Writing – review and editing; data curation; investigation. **Claus Anders Bertelsen:** Investigation; writing – review and editing; formal analysis. **Adam Stearns:** Methodology; investigation; writing – review and editing. **Kristian Storli:** Writing – review and editing; investigation. **Danilo Miskovic:** Conceptualization; methodology; writing – review and editing; writing – original draft; investigation; supervision; resources.

## FUNDING INFORMATION

The authors received no specific funding for this work.

## CONFLICT OF INTEREST STATEMENT

J Fetcher, C Bertelsen, E Van Eetvelde, K Storli, P Lung and D Miskovic have no disclosures to make.

## ETHICS STATEMENT

Ethical approval for the study was obtained from the local research and development department at London Northwest NHS Trust, London.

## Supporting information


File S1.

File S2.

Appendix S1.

## Data Availability

The data that support the findings of this study are available from the corresponding author upon reasonable request.
