# Supplementary material for: Inter‐rater and intra‐rater reliability of multi‐slice CT and three‐dimensional reconstructed imaging analysis of mesenteric vascular anatomy for planning and performing complete mesocolic excision
Source: Colorectal Dis. 2025 Mar 13;27(3):e70025. doi: 10.1111/codi.70025 (PMC11907098; doi:10.1111/codi.70025)
Supplement: Supplementary file 1 — File S1. [file CODI-27-0-s001.pdf]

Complete mesocolic excision anatomical understanding questionaire

https://forms.office.com/r/VSjWb4qgLZ
