# Supplementary material for: Inter‐rater and intra‐rater reliability of multi‐slice CT and three‐dimensional reconstructed imaging analysis of mesenteric vascular anatomy for planning and performing complete mesocolic excision
Source: Colorectal Dis. 2025 Mar 13;27(3):e70025. doi: 10.1111/codi.70025 (PMC11907098; doi:10.1111/codi.70025)
Supplement: Supplementary file 2 — File S2. [file CODI-27-0-s003.pdf]

3DCME: Inter/Intra rater reliability study
Vessel Definitions and Terminology

Vessel classification with review of literature and what is deemed to be most clinically useful. Naming of vessels largely determined by the segment of colon supplied or drained.

The transition between the caecum and ascending colon is delineated by the ileocaecal valve. In lieu of any robust radiological/anatomical precise definitions delineating the hepatic flexure, transverse colon and splenic flexure we leave it to the discretion of each reviewer.

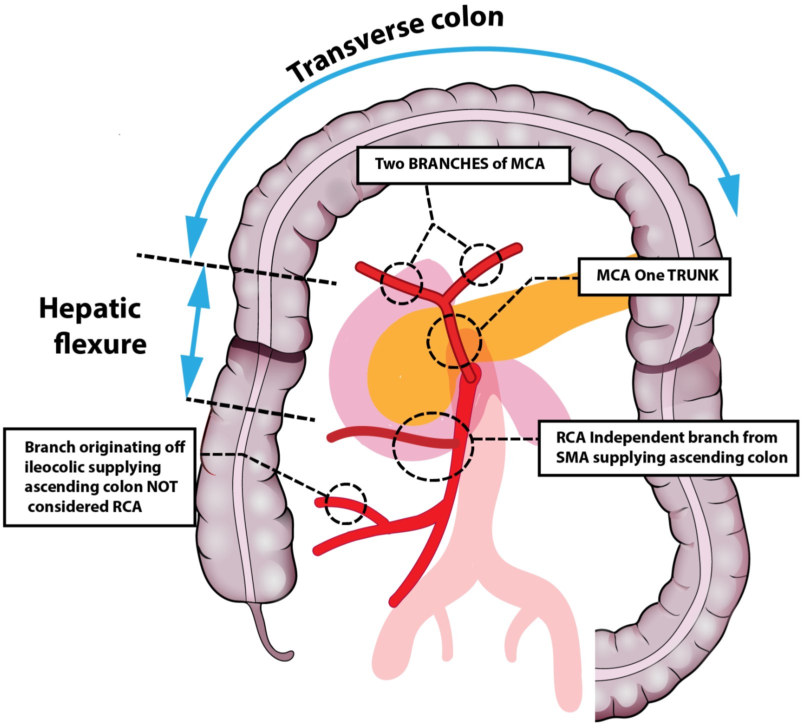

Ileocolic Artery and Vein

Highly consistent vessel present in over 99% of patients. Supplies and drains the caecum and proximal ascending colon

Right Colic Artery

In the literature has a highly varied definition. For this study an Right colic artery is defined as an independent branch arising from the SMA to supply the ascending colon.

Branches arising from the ileocolic artery that supply the ascending colon should not be considered an RCA for the purposes of the study questionnaires.
Branches arising from the ileocolic artery or the middle colic artery that supply the ascending colon should not be considered an RCA for the purposes of the study questionnaires.

Middle Colic Arteries

Middle colic arteries are defined as branches arising from the SMA to supply the transverse colon.

In the typical configuration the MCA will consist of one trunk and will bifurcate into two branches.

Figure two depicts two independent MCA trunks arising from the SMA

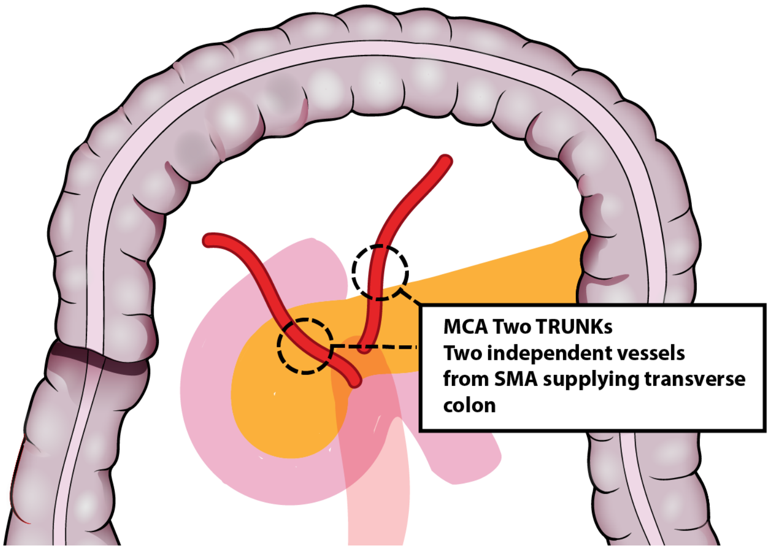

3DCME: Inter/Intra rater reliability study
Vessel Definitions and Terminology

Right Colic Vein

Drains the ascending colon.

Superior Right Colic Vein

Drains the hepatic flexure

Anterior superior pancreatico duodenal vein

Drains the pancreatic head and duodenum

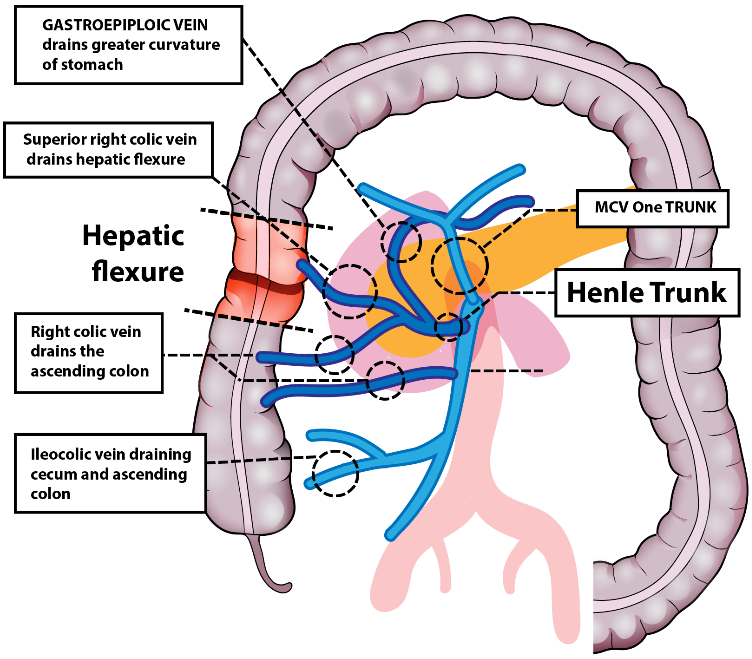

Henles' Trunk

Defined as the confluence of the right gastroepiploic vein (REV) with/without the anterior superior pancreatico-duodenal veins (ASPDV) and one, two or rarely 3-4 colonic veins (right colic vein [RCV], superior right colic vein [SRCV], middle colic vein [MCV] or ileocolic veins [ICV]). Henle is generally present in most cases but may be absent with each vessel draining directly into the SMV as separate vessels.

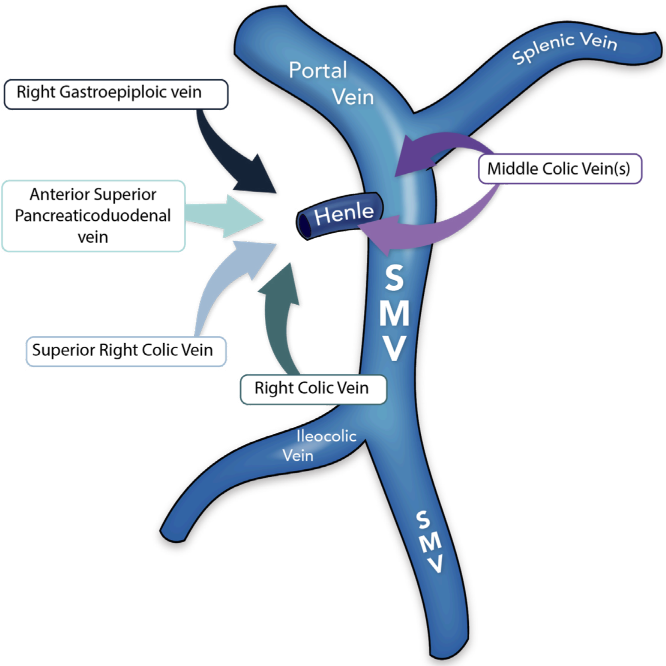

We have classified Henle by the constituent vessels:

1. **Gastro-colic Trunk (GCT)**
   - (RGEV + SRCV)

2. **Gastro-pancreatico Trunk (GPT)**
   - (RGEV + ASPDV)

3. **Gastro-pancreatico-duodenal colic trunk (GPCT)**
   - (RGEV + ASPDV + 1 colic RCV)
   - (RGEV + ASPDV + 1 colic SRCV)
   - (RGEV + ASPDV + 1 colic MCV)
   - (RGEV + ASPDV + 2 colic SRCV + RCV)
   - (RGEV + ASPDV + 2 colic SRCV + MCV)
   - (RGEV + ASPDV + 2 colic RCV + MCV)
   - (RGEV + ASPDV + 2 colic RCV + ICV)
   - (RGEV + ASPDV + 3 colic RCV + SRCV +MCV)
   - (RGEV + ASPDV + 3 colic RCV + SRCV +MCV)
   - (RGEV + ASPDV + 3 colic RCV + SRCV + ICV)

4. **Colo-pancreatico Trunk (CPT)**
   - (ASPDV + SRCV)

3DCME: Inter/Intra rater reliability study
Vessel Definitions and Terminology

Middle colic veins

Vessels draining the transverse colon. The trunk of the MCV is defined as the main stem of the vessel which drains into a larger tributary (e.g. SMV, splenic vein, Henle etc.)

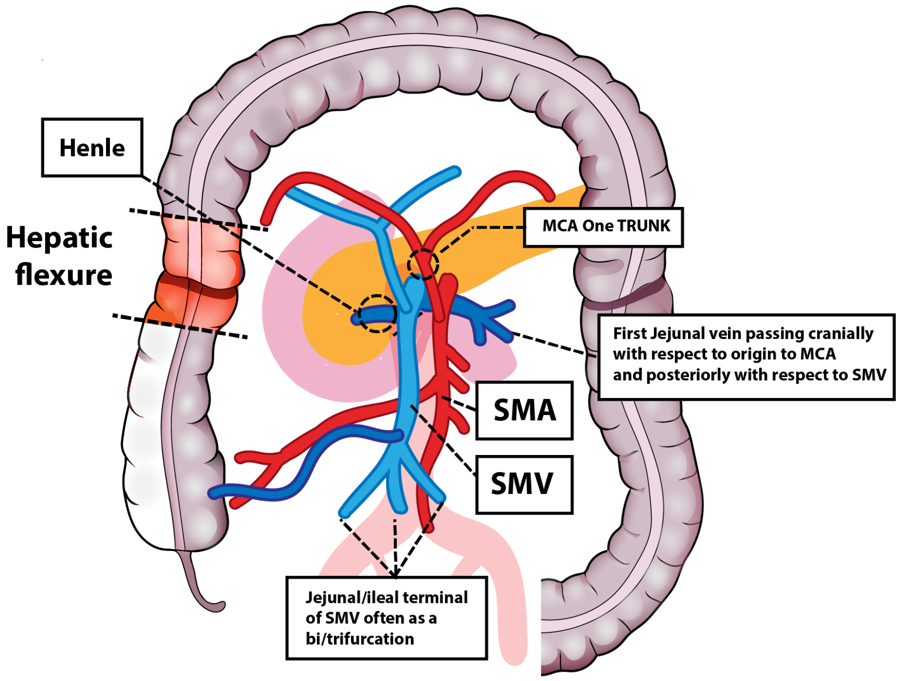

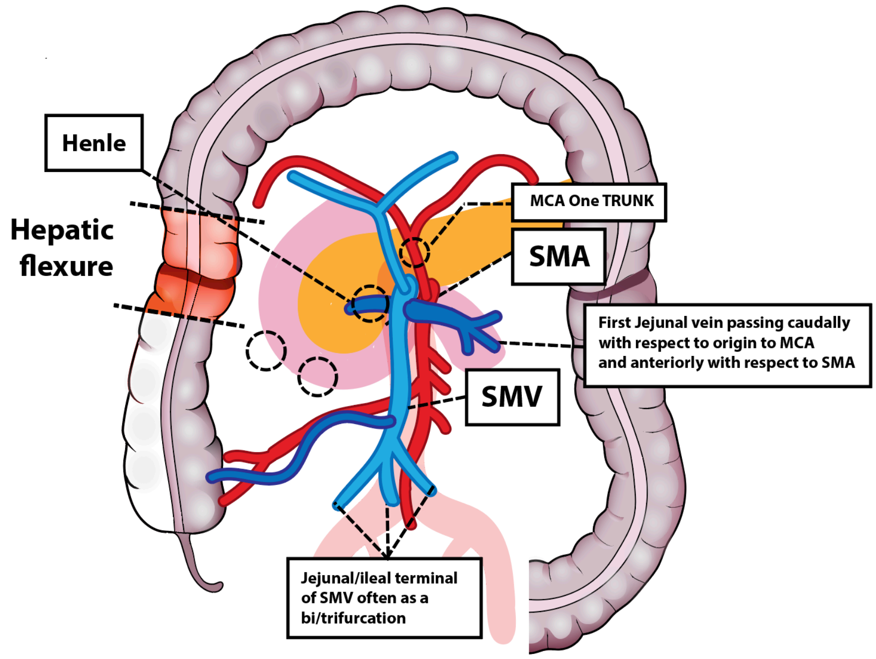

First jejunal vein

First jejunal branch is defined as the most cranial branch draining proximal jejunal loops into the SMV.

The first jejunal branch can be classified as either posterior or anterior depending on its position with relation to the SMA.

The figures also depict its relation to the origin MCA; either cranial (above) or caudal (below).
