# Supplementary material for: Inter‐rater and intra‐rater reliability of multi‐slice CT and three‐dimensional reconstructed imaging analysis of mesenteric vascular anatomy for planning and performing complete mesocolic excision
Source: Colorectal Dis. 2025 Mar 13;27(3):e70025. doi: 10.1111/codi.70025 (PMC11907098; doi:10.1111/codi.70025)
Supplement: Supplementary file 3 — Appendix S1. [file CODI-27-0-s002.docx]

Supplemental Data

Supplementary file 1: link to online CME anatomy questionnaire.

Supplementary file 2: Mesenteric vessel definitions.


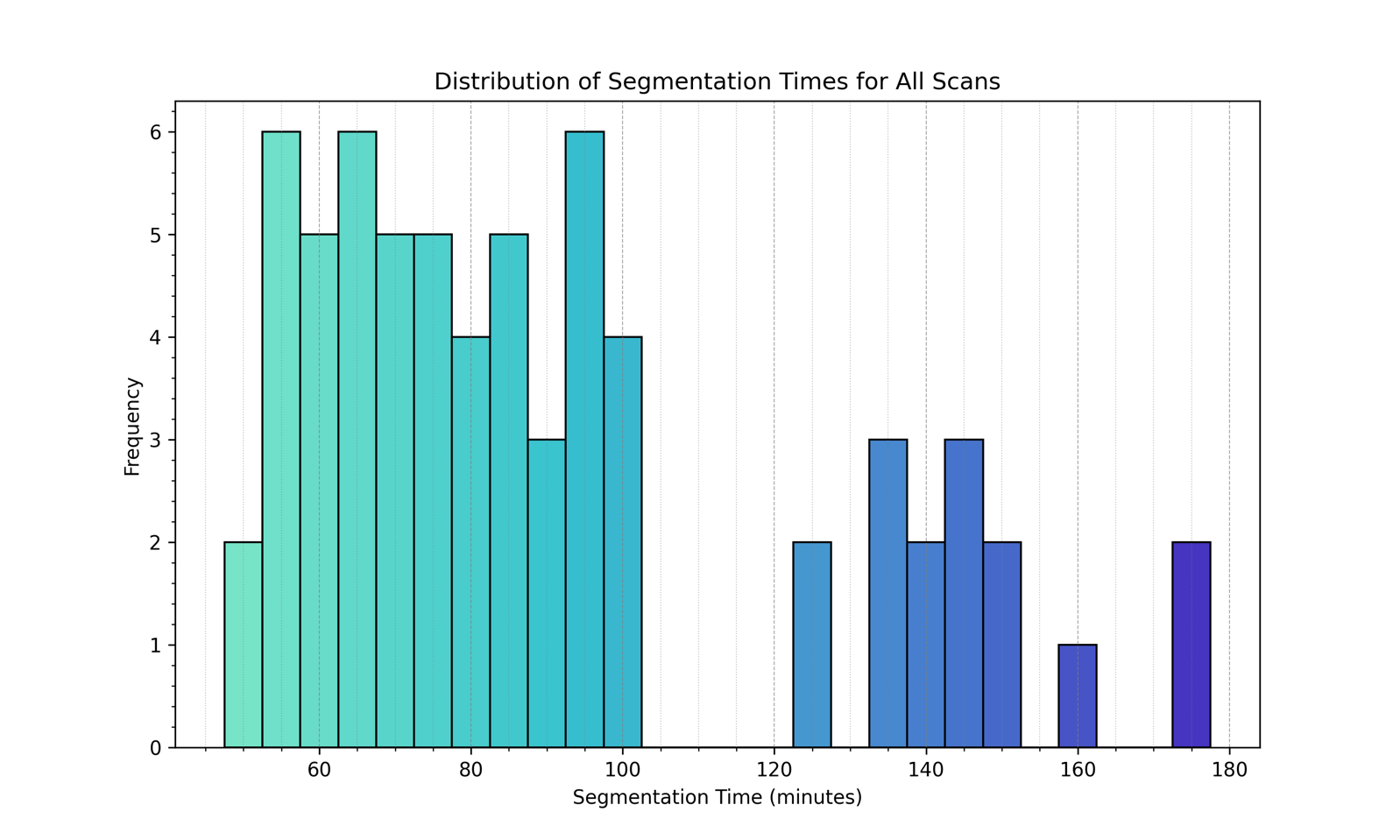


**Supplementary Figure 1**: Distribution of Time Required for 3D Model Generation. Histogram showing the frequency of cases by segmentation duration (minutes).

­­­
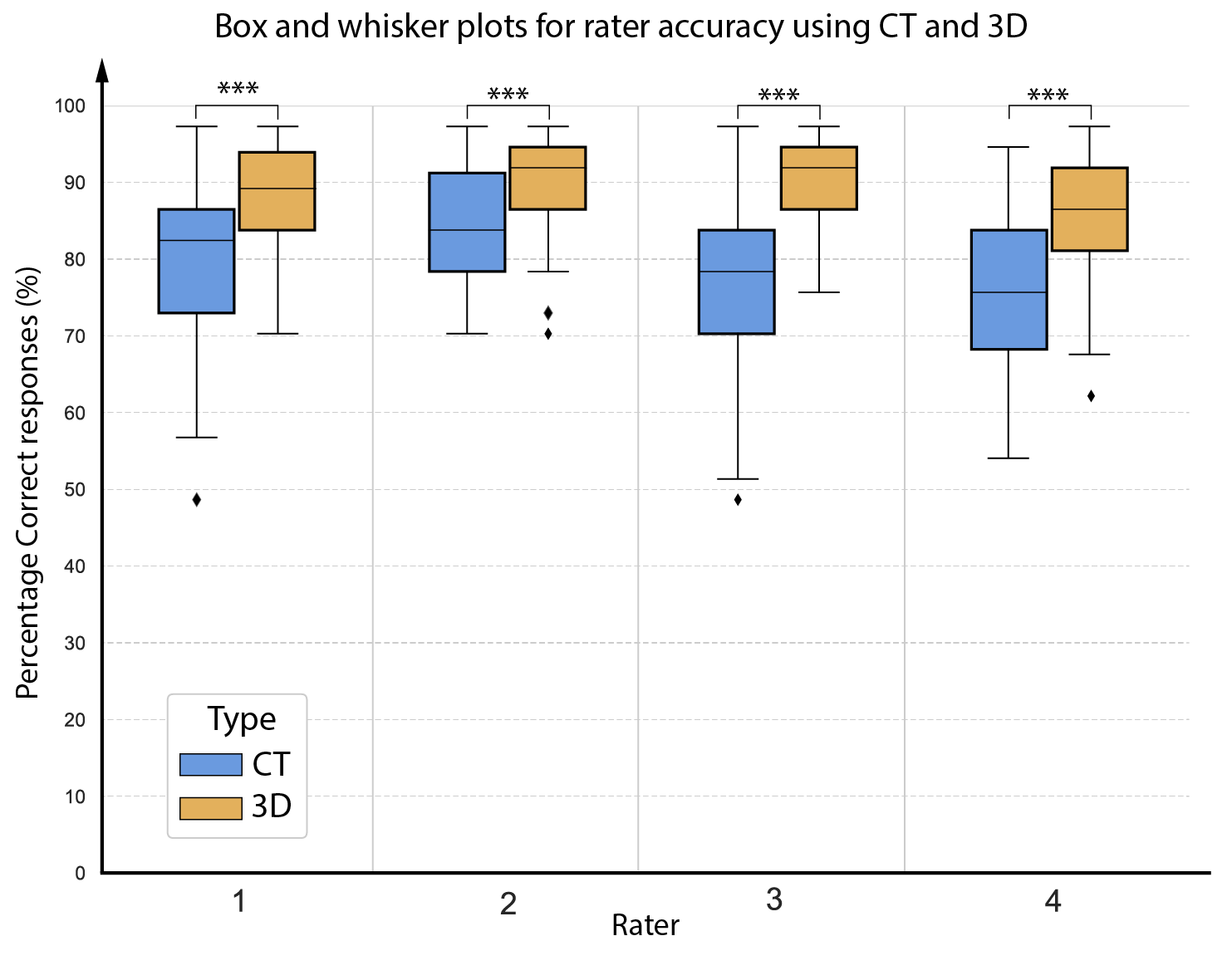


**Supplementary Figure 2:** Box Plot Distribution of Rater Accuracy Using CT and 3D Imaging Modalities. Correct response for raters 1-4. For each rater there is a pair of box plots where the blue box represents the distribution of correct responses when using CT imaging, and the orange box corresponds to 3D imaging. The central line in each box indicates the median accuracy, the box limits represent the interquartile range (IQR), and the whiskers extend to the furthest point within 1.5 times the IQR from the box. Outliers are represented as individual points outside the whiskers.


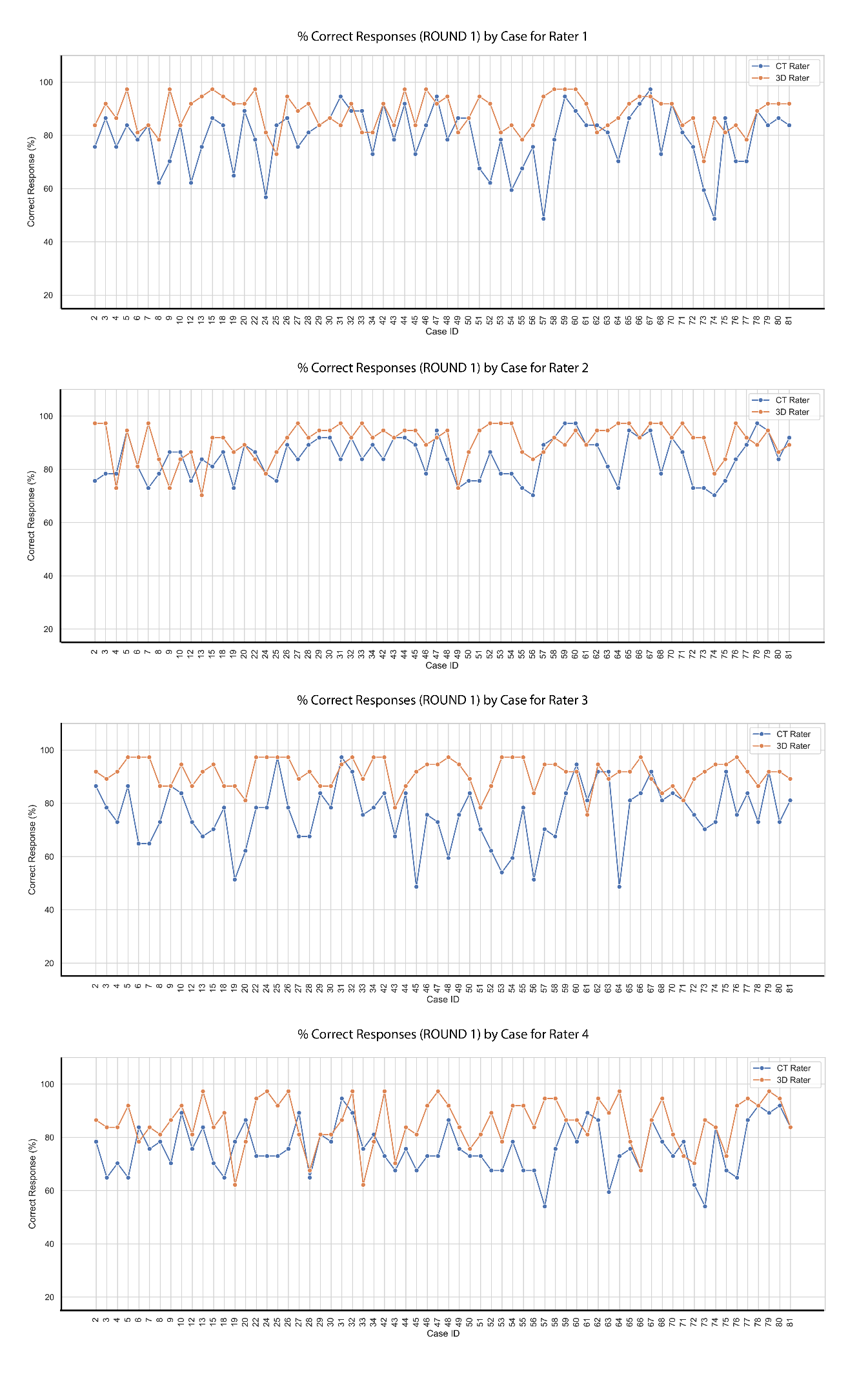


**Supplementary Figure 3:** Individual Rater Accuracy Comparison by Case in ROUND 1 Using CT and 3D Models. This series of line graphs illustrates the percentage of correct responses for each case by four separate raters in the second evaluation round. The accuracy is compared between two diagnostic tools: CT imaging (depicted in blue) and 3D modelling (depicted in orange). Each graph corresponds to one of the four raters, with the x-axis representing individual cases and the y-axis showing the accuracy percentage. Data points on the lines indicate the accuracy achieved by the respective rater for each case using the specified imaging modality.


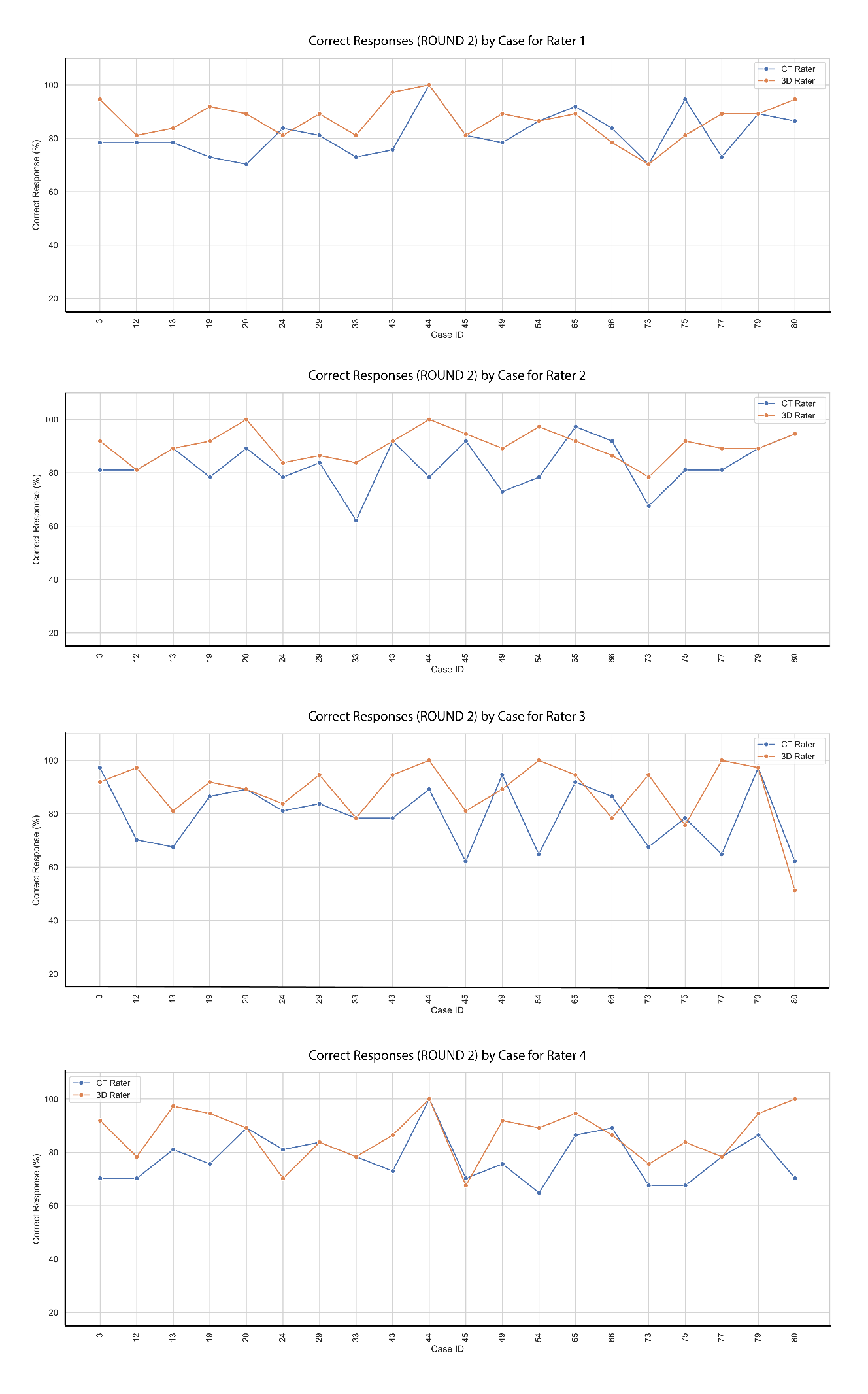


**Supplementary Figure 4:** Individual Rater Accuracy Comparison by Case in ROUND 2 Using CT and 3D Models. This series of line graphs illustrates the percentage of correct responses for each case by four separate raters in the second evaluation round. The accuracy is compared between two diagnostic tools: CT imaging (depicted in blue) and 3D modelling (depicted in orange). Each graph corresponds to one of the four raters, with the x-axis representing individual cases and the y-axis showing the accuracy percentage. Data points on the lines indicate the accuracy achieved by the respective rater for each case using the specified imaging modality.


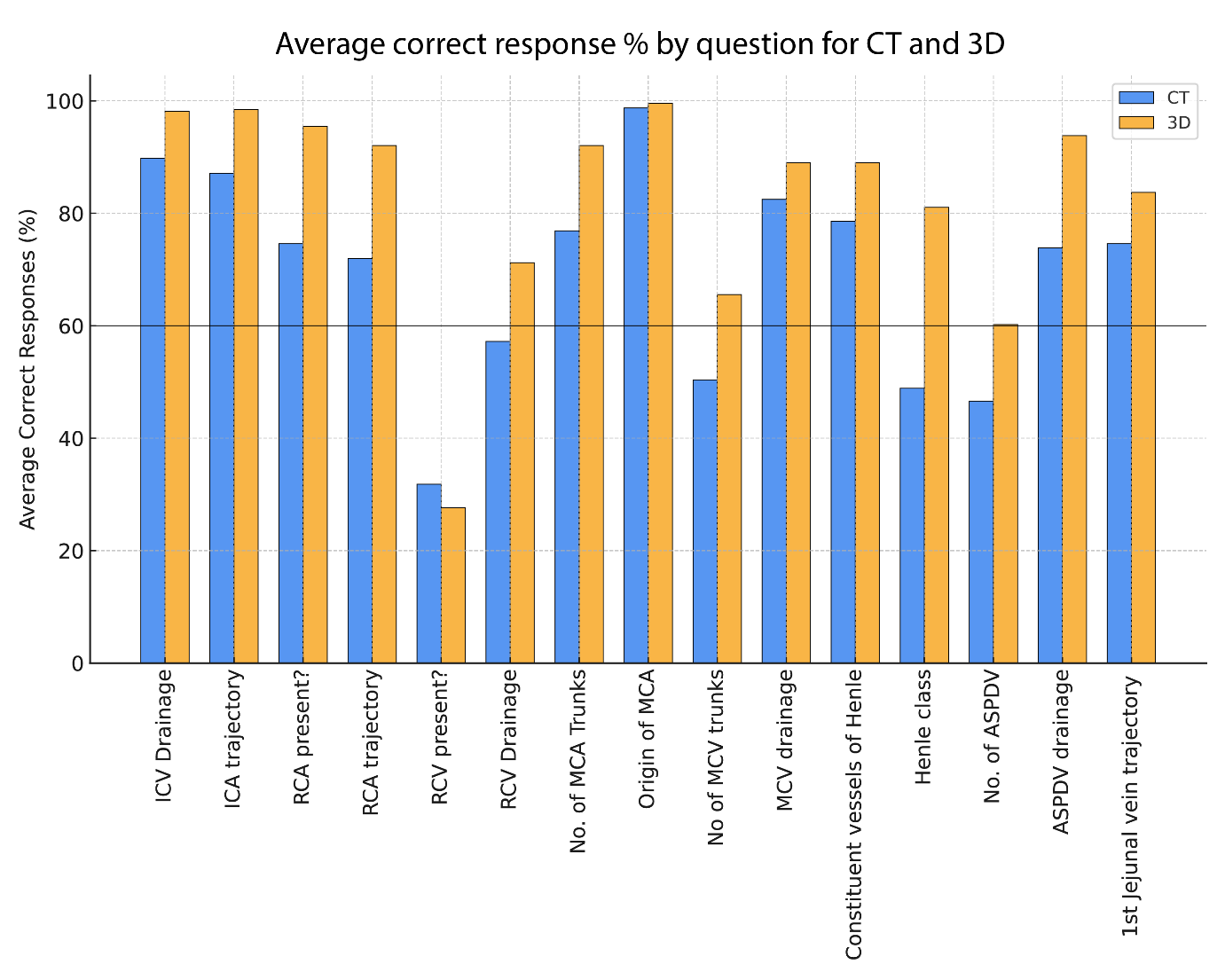


**Supplementary Figure 5:** Comparison of Vessel Identification Accuracy: CT vs 3D Models. Average percentage of correct responses across all raters for individual mesenteric vessels, comparing conventional CT scans (blue) with 3D reconstructions (orange).


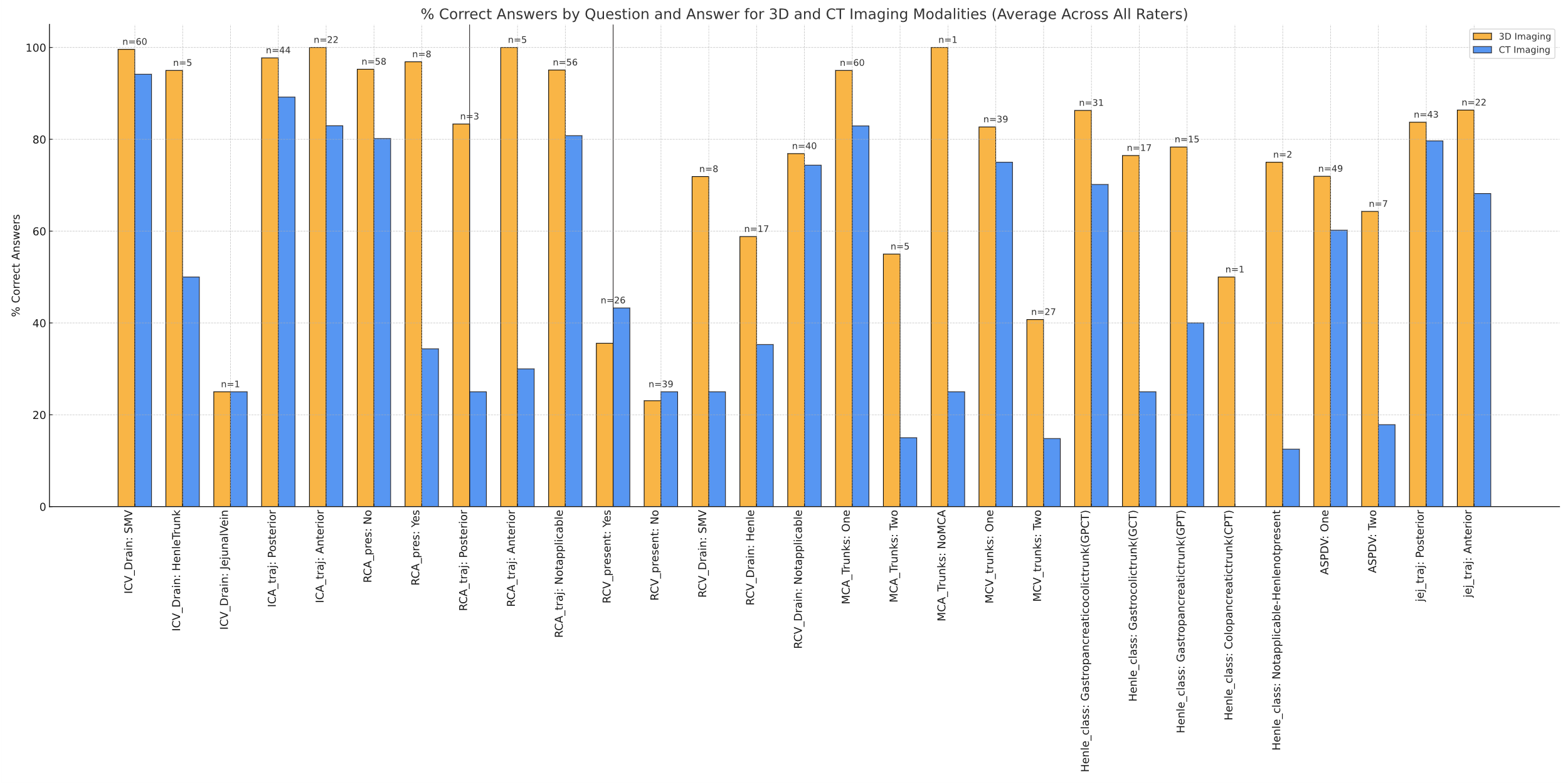


**Supplementary Figure 6**: Comparative Analysis of Correct Response Rates by Imaging Modality Across Anatomical Variations. This paired bar chart displays the mean percentage of correct diagnoses by all evaluators, contrasting results obtained using 3D imaging (orange bars) with those from CT imaging (blue bars). Each pair of bars corresponds to a different anatomical variation as specified on the x-axis. The number of cases (n) featuring each anatomical variant is indicated atop the respective bars, reflecting the sample size for each question category.

| **Question** | **Answer** | **Total** | **RATER 1** | | | | **RATER 2** | | | | **RATER 3** | | | | **RATER 4** | | | |
| --- | --- | --- | --- | --- | --- | --- | --- | --- | --- | --- | --- | --- | --- | --- | --- | --- | --- | --- |
|  |  |  | **3D** | | **CT** | | **3D** | | **CT** | | **3D** | | **CT** | | **3D** | | **CT** | |
|  |  |  | **n** | **%** | **n** | **%** | **n** | **%** | **n** | **%** | **n** | **%** | **n** | **%** | **n** | **%** | **n** | **%** |
| ICV  drainage | SMV | 60 | 60 | 100.00 | 57 | 95.00 | 60 | 100.00 | 60 | 100.00 | 59 | 98.33 | 51 | 85.00 | 60 | 100.00 | 58 | 96.67 |
|  | HenleTrunk | 5 | 4 | 80.00 | 3 | 60.00 | 5 | 100.00 | 5 | 100.00 | 5 | 100.00 | 2 | 40.00 | 5 | 100.00 | 0 | 0.00 |
|  | JejunalVein | 1 | 0 | 0.00 | 0 | 0.00 | 0 | 0.00 | 1 | 100.00 | 1 | 100.00 | 0 | 0.00 | 0 | 0.00 | 0 | 0.00 |
| ICA  trajectory | Posterior | 44 | 42 | 95.45 | 40 | 90.91 | 44 | 100.00 | 44 | 100.00 | 44 | 100.00 | 36 | 81.82 | 42 | 95.45 | 37 | 84.09 |
|  | Anterior | 22 | 22 | 100.00 | 18 | 81.82 | 22 | 100.00 | 22 | 100.00 | 22 | 100.00 | 19 | 86.36 | 22 | 100.00 | 14 | 63.64 |
| RCA  present? | No | 58 | 56 | 96.55 | 53 | 91.38 | 57 | 98.28 | 50 | 86.21 | 56 | 96.55 | 34 | 58.62 | 52 | 89.66 | 49 | 84.48 |
|  | Yes | 8 | 8 | 100.00 | 2 | 25.00 | 8 | 100.00 | 4 | 50.00 | 8 | 100.00 | 4 | 50.00 | 7 | 87.50 | 1 | 12.50 |
| RCA  Trajectory | Posterior | 3 | 2 | 66.67 | 0 | 0.00 | 3 | 100.00 | 2 | 66.67 | 3 | 100.00 | 1 | 33.33 | 2 | 66.67 | 0 | 0.00 |
|  | Anterior | 5 | 5 | 100.00 | 1 | 20.00 | 5 | 100.00 | 2 | 40.00 | 5 | 100.00 | 3 | 60.00 | 5 | 100.00 | 0 | 0.00 |
|  | N/A | 56 | 54 | 96.43 | 54 | 96.43 | 55 | 98.21 | 48 | 85.71 | 54 | 96.43 | 32 | 57.14 | 50 | 89.29 | 47 | 83.93 |
| RCV  present? | Yes | 26 | 6 | 23.08 | 7 | 26.92 | 5 | 19.23 | 14 | 53.85 | 8 | 30.77 | 8 | 30.77 | 18 | 69.23 | 16 | 61.54 |
|  | No | 39 | 14 | 35.90 | 22 | 56.41 | 8 | 20.51 | 8 | 20.51 | 9 | 23.08 | 7 | 17.95 | 5 | 12.82 | 2 | 5.13 |
| RCV  drainage | SMV | 8 | 6 | 75.00 | 2 | 25.00 | 6 | 75.00 | 4 | 50.00 | 6 | 75.00 | 0 | 0.00 | 5 | 62.50 | 2 | 25.00 |
|  | Henle | 17 | 13 | 76.47 | 11 | 64.71 | 14 | 82.35 | 8 | 47.06 | 11 | 64.71 | 4 | 23.53 | 2 | 11.76 | 1 | 5.88 |
|  | N/A | 40 | 26 | 65.00 | 17 | 42.50 | 31 | 77.50 | 32 | 80.00 | 31 | 77.50 | 32 | 80.00 | 35 | 87.50 | 38 | 95.00 |
| No. MCA  Trunks | One | 60 | 57 | 95.00 | 58 | 96.67 | 54 | 90.00 | 52 | 86.67 | 57 | 95.00 | 46 | 76.67 | 60 | 100.00 | 43 | 71.67 |
|  | Two | 5 | 4 | 80.00 | 1 | 20.00 | 4 | 80.00 | 0 | 0.00 | 3 | 60.00 | 1 | 20.00 | 0 | 0.00 | 1 | 20.00 |
|  | No MCA | 1 | 1 | 100.00 | 0 | 0.00 | 1 | 100.00 | 0 | 0.00 | 1 | 100.00 | 0 | 0.00 | 1 | 100.00 | 1 | 100.00 |
| No MCV  Trunks | One | 39 | 34 | 87.18 | 34 | 87.18 | 32 | 82.05 | 31 | 79.49 | 30 | 76.92 | 22 | 56.41 | 33 | 84.62 | 30 | 76.92 |
|  | Two | 27 | 11 | 40.74 | 1 | 3.70 | 13 | 48.15 | 8 | 29.63 | 16 | 59.26 | 7 | 25.93 | 4 | 14.81 | 0 | 0.00 |
| Henle class | GPCT | 31 | 28 | 90.32 | 25 | 80.65 | 28 | 90.32 | 25 | 80.65 | 28 | 90.32 | 20 | 64.52 | 23 | 74.19 | 17 | 54.84 |
|  | GCT | 17 | 13 | 76.47 | 1 | 5.88 | 12 | 70.59 | 1 | 5.88 | 13 | 76.47 | 5 | 29.41 | 14 | 82.35 | 10 | 58.82 |
|  | GPT | 15 | 11 | 73.33 | 4 | 26.67 | 14 | 93.33 | 11 | 73.33 | 13 | 86.67 | 7 | 46.67 | 9 | 60.00 | 2 | 13.33 |
|  | CPT | 1 | 0 | 0.00 | 0 | 0.00 | 1 | 100.00 | 0 | 0.00 | 1 | 100.00 | 0 | 0.00 | 0 | 0.00 | 0 | 0.00 |
|  | N/A | 2 | 2 | 100.00 | 0 | 0.00 | 2 | 100.00 | 1 | 50.00 | 2 | 100.00 | 0 | 0.00 | 0 | 0.00 | 0 | 0.00 |
| No. ASPDV | One | 49 | 34 | 69.39 | 21 | 42.86 | 38 | 77.55 | 37 | 75.51 | 37 | 75.51 | 33 | 67.35 | 32 | 65.31 | 27 | 55.10 |
|  | Two | 7 | 4 | 57.14 | 2 | 28.57 | 6 | 85.71 | 1 | 14.29 | 4 | 57.14 | 1 | 14.29 | 4 | 57.14 | 1 | 14.29 |
| Jejunal vein  trajectory | Posterior | 43 | 36 | 83.72 | 35 | 81.40 | 39 | 90.70 | 38 | 88.37 | 42 | 97.67 | 31 | 72.09 | 27 | 62.79 | 33 | 76.74 |
|  | Anterior | 22 | 18 | 81.82 | 15 | 68.18 | 21 | 95.45 | 18 | 81.82 | 19 | 86.36 | 14 | 63.64 | 18 | 81.82 | 13 | 59.09 |

**Supplementary Table 1:** Rater-Specific Diagnostic Accuracy for Anatomical Variants Using CT and 3D Imaging. This table presents a detailed breakdown of the correct response rates for each rater, across various anatomical variants, using both CT and 3D imaging modalities. The 'Total' column indicates the number of cases for each anatomical feature, while subsequent columns provide counts (n) and percentages (%) of correct diagnoses by each rater for the two imaging methods.


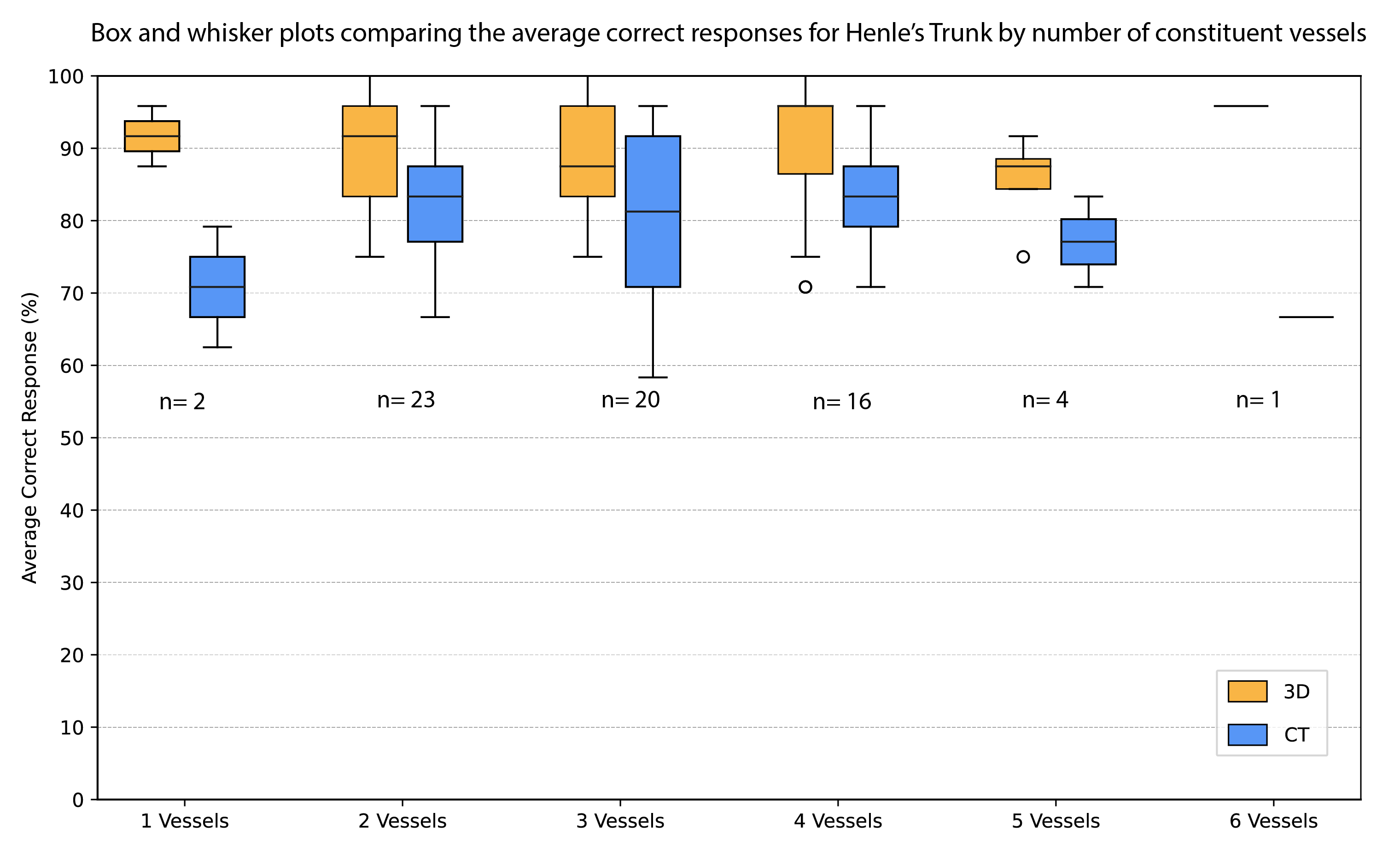


**Supplementary Figure 7**: Box and whisker plots comparing the average correct responses for Henle's trunk using 3D and CT imaging modality by the number of constituent vessels. There was no statistical difference found between the magnitude of the improvement of 3D over CT for the number of vessels as assessed by Kruskall-Wallis test.
